# Circulating Levels of MicroRNA from Children with Newly Diagnosed Type 1 Diabetes and Healthy Controls: Evidence That miR-25 Associates to Residual Beta-Cell Function and Glycaemic Control during Disease Progression

**DOI:** 10.1155/2012/896362

**Published:** 2012-07-05

**Authors:** Lotte B. Nielsen, Cheng Wang, Kaspar Sørensen, Claus H. Bang-Berthelsen, Lars Hansen, Marie-Louise M. Andersen, Philip Hougaard, Anders Juul, Chen-Yu Zhang, Flemming Pociot, Henrik B. Mortensen

**Affiliations:** ^1^Department of Pediatrics, Herlev Hospital, 2730 Herlev, Denmark; ^2^Faculty of Health Sciences, University of Copenhagen, 2200 Copenhagen, Denmark; ^3^Jiangsu Engineering Research Center for microRNA Biology and Biotechnology, State Key Laboratory of Pharmaceutical Biotechnology, School of Life Sciences, Nanjing University, Nanjing 210093, China; ^4^Department of Growth and Reproduction, Rigshospitalet and Faculty of Health Sciences, University of Copenhagen, 2100 Copenhagen, Denmark; ^5^Glostrup Research Institute, Glostrup Hospital and Center for Non-Coding RNA in Technology and Health, University of Copenhagen, 2600 Glostrup, Denmark; ^6^Department of Statistics, University of Southern Denmark, 5000 Odense, Denmark

## Abstract

This study aims to identify key miRNAs in circulation, which predict ongoing beta-cell destruction and regeneration in children with newly diagnosed Type 1 Diabetes (T1D). We compared expression level of sera miRNAs from new onset T1D children and age-matched healthy controls and related the miRNAs expression levels to beta-cell function and glycaemic control. Global miRNA sequencing analyses were performed on sera pools from two T1D cohorts (*n* = 275 and 129, resp.) and one control group (*n* = 151). We identified twelve upregulated human miRNAs in T1D patients (miR-152, miR-30a-5p, miR-181a, miR-24, miR-148a, miR-210, miR-27a, miR-29a, miR-26a, miR-27b, miR-25, miR-200a); several of these miRNAs were linked to apoptosis and beta-cell networks. Furthermore, we identified miR-25 as negatively associated with residual beta-cell function (est.: −0.12, *P* = 0.0037), and positively associated with glycaemic control (HbA1c) (est.: 0.11, *P* = 0.0035) 3 months after onset. In conclusion this study demonstrates that miR-25 might be a “tissue-specific” miRNA for glycaemic control 3 months after diagnosis in new onset T1D children and therefore supports the role of circulating miRNAs as predictive biomarkers for tissue physiopathology and potential intervention targets.

## 1. Introduction

As an autoimmune disease-type 1 diabetes (T1D) results from an immune-mediated destruction of the insulin producing beta-cells reflected by the appearance of the pancreatic autoantibodies. The destruction of these cells implies a progressive, irreversible loss of the endogenous insulin production, leading to daily treatment with exogenous insulin. 

At time of diagnosis a child with T1D is estimated to have lost approx.  80–90% of the insulin producing beta-cell function/mass. Shortly after the initial insulin treatment, several children experience a period of increased endogenous insulin production followed by a reduced need of exogenous insulin, referred to as the remission phase [1]. The regenerative potential that exists within this time interval particularly in children gives a unique possibility of intervention treatment, that is, with beta-cell growth factors to maintain the individual insulin production, leading to improved glycaemic control and fewer complications in eyes, kidneys, and nerves. 

The hypothesis of the study is that potential new biomarkers (miRNAs and/or miRNA patterns) in serum from children with newly diagnosed T1D can predict destruction or regeneration of the endogenous residual beta-cell function. miRNAs are small noncoding RNAs involved in posttranscriptional regulation of protein translation either through mRNA destabilization or inhibition [2, 3]. miRNAs are found in solid tissues and cell culture samples, and several studies confirm their presences in body fluids as blood, saliva, urine, and serum [4–7]. The stability of serum miRNAs has been investigated under harsh conditions including boiling, low/high pH, extended storage, and freeze-thaw cycles without any significant difference compared to nontreated serum samples [8]. Taken together these results show that serum miRNAs, are stable and that they may reflect cellular dysfunctions in various chronic diseases. In addition, several miRNAs clearly have a role in metabolic pathways for example, miR-33a/b inhibition in nonhuman primates raises plasma HDL cholesterol and lowers triglycerides [9], and silencing of miR103/107 seems to have beneficial effects on insulin sensitivity in obese mice possibly through its target gene caveolin-1 which is a critical regulator of the insulin receptor [10]. 

The objective of the current study was therefore to identify new biomarkers (key miRNAs/miRNA patterns), which are predictive for destruction or regeneration of the endogenous residual beta-cell function by investigating miRNAs in serum samples from new-onset T1D patients and age-matched controls.

## 2. Methods 

### 2.1. Study Cohorts

Serum samples from two unique T1D cohorts and one control group were analysed for miRNA expression.

### 2.2. International Remission Phase Cohort (The Hvidoere Cohort)

The Hvidoere Remission Phase cohort is a longitudinally, observational cohort of newly diagnosed T1D children collected through 18 paediatric centres primarily in Europe [11]. Blood samples for centrally determined HbA1c, stimulated C-peptide, glucagon, incretin hormones, cytokines, immunology, and DNA were collected prospectively at a meal-stimulated test 1, 6, and 12 months after diabetes onset. 275 patients were included in the study.

### 2.3. The Danish Remission Cohort

The Danish Remission Phase cohort includes new onset T1D children treated at four Danish paediatric centres. The study design is analogous to the Hvidoere cohort, except for the inclusion of a three-month meal-stimulated test. 129 children were included in the study [12].

### 2.4. Healthy Control Children (The Copenhagen Puberty Study)

Healthy controls consist of girls and boys between 6.7 and 13.7 years, recruited from public schools in the Copenhagen area as part of a mixed cross-sectional and longitudinal study, the COPENHAGEN Puberty Study [13, 14]. Information on gender, age, pubertal stage, height, and weight was recorded. Blood samples were analysed for metabolic parameters (glucose, insulin, cholesterol, and triglycerides). 151 children were included in the present study. 

### 2.5. miRNA Purification, Solexa Sequencing, and Quantitative RT-PCR

The work flow was separated into three steps: (i) initial screening by high-throughput Solexa sequencing using pooled serum samples, (ii) qRT-PCR validation in a large number of individual serum samples arranged in multiple training and testing sets, and (iii) statistical evaluation of the diagnostic or prognostic value of the serum miRNA profiling system. 

#### 2.5.1. Serum Pools and miRNA Purification

Serum pools were prepared from all three cohorts. From the Hvidoere cohort 100 *μ*L serum from 275 children was pooled, from the Danish cohort 200 *μ*L serum from 129 children was pooled, and finally 200 *μ*L serum from 151 healthy control children was pooled. Total RNA was extracted from serum pools using TRIzol Reagent (Invitrogen, Carlsbad, CA, USA) according to the manufacturer's instructions. Furthermore, total RNA from all individuals in the three cohorts (the Hvidoere, the Danish, and healthy controls) was isolated and stored at −80°C for later testing of candidate miRNAs.

#### 2.5.2. Solexa Sequencing

The pooled samples from the 3 cohorts (a total of 5–10 *μ*g RNA) were sequenced on the Solexa sequencing platform (Illumina). After PAGE purification of small RNA molecules under 30 bases and ligation of a pair of Solexa adaptors to their 5′ and 3′ ends, the small RNA molecules were amplified using the adaptor primers for 17 cycles, and fragments around 90 bp (small RNA+adaptors) were isolated from agarose gel.  The purified DNA was used directly for cluster generation and sequencing analysis using the Illumina Genome Analyzer IIx according to the manufacturer's instructions. Image files were generated by the sequencer and were processed to produce digital-quality data. After masking the adaptor sequences and the removal of contaminated reads, clean reads were processed for *in silico* analysis as previously described [8]. After comparing the serum miRNA profile in the control group versus the patients, a panel of differentially (as defined by copy number >100 and 2-fold altered expression) expressed miRNAs was derived. 

#### 2.5.3. Quantitative RT-PCR of Mature miRNA

Quantification of mature miRNAs was carried out using Taqman miRNA probes (Applied Biosystems, Foster City, CA, USA) according to the manufacturer's instructions. Briefly, 2 *μ*L of total RNA is reverse-transcribed to cDNA using AMV reverse transcriptase and a stem-loop RT primer (Applied Biosystems). Real-time PCR was performed using a TaqMan PCR kit on an Applied Biosystems 7300 Sequence Detection System (Applied Biosystems). All reactions, including non-template controls, were run in triplicate. Twenty-four candidate miRNAs were reanalysed by qRT-PCR analyses on individual samples. We examined the expression levels of the candidate miRNAs in a subset of the cohorts consisting of 108 patients (1-month samples from the Danish Remission Phase cohort and 12-month samples from the Hvidoere cohort) and 54 controls. 

### 2.6. Statistics

For the cohort comparisons each miRNA was studied separately as function of cohort, age, and sex on a logarithmic scale. All comparisons were adjusted for multiple testing by Hochberg approach. 

Statistical evaluation of miRNA association to disease progression (endpoints: stimulated C-peptide and HbA1c) was done by multiple linear regression analysis adjusting for age and sex. miRNA expression was presented as 2^(−ΔCt)^ transformed values (the difference in Ct value between a given miRNA and the reference miRNA (a combination of three miRNAs of the let-7 family); all values represent the geometricmean of triplicates measures). Stimulated C-peptide was studied on logarithmic scale to obtain normal distribution, while HbA1c was normally distributed. The results were interpreted as the change in actual HbA1c values (%) and the percentage change in stimulated C-peptide corresponding to the range between the 25 percentile and the 75 percentile (interquartile range) of the ΔCt values of the relevant miRNA. Data were analysed using SAS (version 9.2, SAS Institute; Cary, NC). A *P*-value of <0.05 was considered statistically significant.

### 2.7. Ethical Approval

Ethical approval has been obtained for the Hvidoere and the Danish Remission Phase Studies allowing the samples to be analyzed for miRNAs (KA 99063 and KA 04010gm, resp.). The COPENHAGEN Puberty Study (ClinicalTrialsGov ID NCT01411527) including additional genetic and epigenetic studies was approved by the ethical committee (KF 01 282212 and V200.1996/90). 

## 3. Results

### 3.1. Comparing Serum miRNAs Expression Levels between Two Paediatric Type 1 Diabetes Cohorts and an Age-Matched Control Group

**Figure 1:**
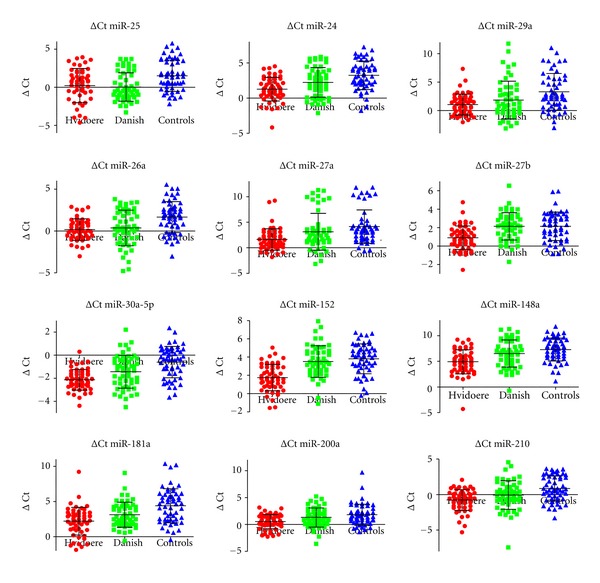
Twelve miRNAs were differentially expressed between the diabetes cohorts and the controls. ΔCt values are plotted for each cohort (Hvidoere (red), Danish (green) and controls (blue)). The bars represent geometric means of the ΔCt values ± SD.

miRNA was purified from a pool of serum from each cohort (serum samples were taken 1 month after diagnosis for both diabetes cohorts), and the presence and levels of miRNAs were identified by global Solexa sequencing. We identified in total 240 different miRNAs from these cohorts; this corresponds to approx. 15% of all known human miRNAs (miRbase18 identifies 1527 human miRNAs). 47 miRNAs fulfilled our criteria for differential expression between the diabetes cohorts and the control group (>100 copy number and 2-fold altered expression). Of these 24 miRNAs (miR-103, miR-10a, miR-125b, miR-134, miR-199a, miR-200c, miR-21, miR-26b, miR-29b, miR-340, miR-320a, miR-222, miR-152, miR-30a-5p, miR-181a, miR-24, miR-148a, miR-210, miR-27a, miR-29a, miR-26a, miR-27b, miR-25, and miR-200a) were selected for further analyses by qRT-PCR in a subset (*n* = 54) of samples from each cohort. Consistently we found all 24 miRNAs upregulated by both Solexa sequencing and qPCR analyses. A combination of three miRNAs of the let-7 family was used as reference for normalisation of miRNA expression levels measured by qRT-PCR. By regression analysis adjusting for age, sex, and multiple testing we found 12 miRNAs (miR-152, miR-30a-5p, miR-181a, miR-24, miR-148a, miR-210, miR-27a, miR-29a, miR-26a, miR-27b, miR-25, and miR-200a) to be significantly differentially expressed between either both diabetes cohorts and the control group or just one of the diabetes cohorts and the controls (Table 1) (*P* < 0.05). The ΔCt values of these 12 miRNAs are presented in Figure 1. 

### 3.2. Association between Qualified miRNAs and Disease Progression in Children with New Onset Type 1 Diabetes

**Figure 2:**
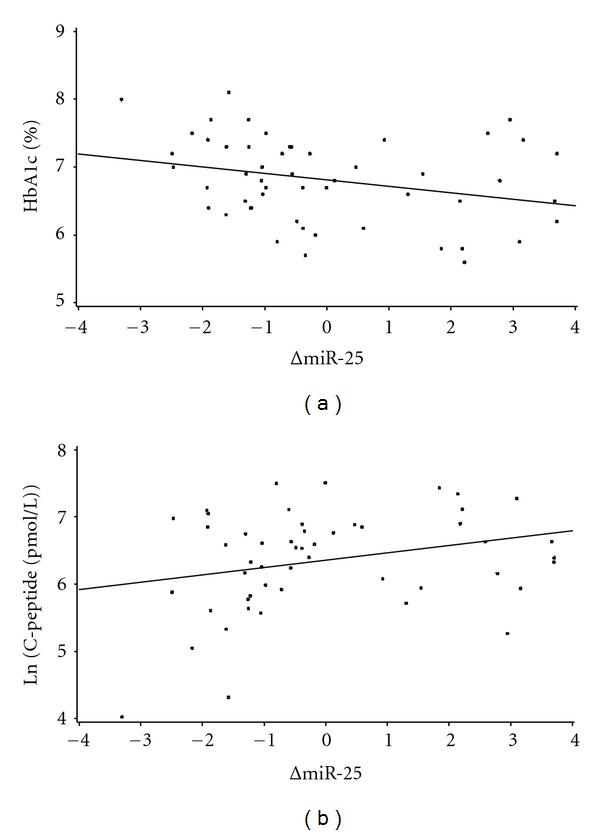
(a) A significant negative association between miR-25 one month after diagnosis and HbA1c (%) was shown at 3 months in the Danish Remission Phase Cohort (*P* = 0.003) while (b) stimulated C-peptide (pmol/L) was positively associated to miR-25 at the same time point (*P *= 0.0045).

The qualified miRNAs with differential expression pattern between cases and controls were analysed by qRT-PCR analyses for association with disease progression in the two diabetes cohorts. Disease progression was described by glycaemic control as assessed by HbA1c or residual beta-cell function estimated by stimulated C-peptide. In the Danish cohort miR-25 measured at 1 month after disease onset was negatively associated with the HbA1c level and positively associated with stimulated C-peptide levels 3 months after onset. The regression analyses suggest a 0.22% decrease in HbA1c level (0.08–0.37) (*P *= 0.0035) and a 22.4% (8.3–34.3) (*P* = 0.0037) increase in stimulated C-peptide between the 25–75 interquartile range of ΔmiR-25 (Figures 2(a) and 2(b)). miR-25 measured at 12 months after disease onset was not associated with stimulated C-peptide or HbA1c in the Hvidoere cohort (data not shown). Furthermore, we analysed for association between the 12 qualified miRNAs and the pancreatic autoantibodies (GAD, IA, IA-2A and ZnT8Ab) (data not shown). We found no association between these parameters in any of the cohorts. 

## 4. Discussion

This is the first study to compare miRNA levels in serum samples from children with or without T1D. Serum samples from approximately 400 new onset T1D children and 150 healthy age-matched controls were analysed. Twelve differentially expressed miRNAs between cases and controls were identified. Several of these miRNAs are involved in regulation of apoptosis (miR-181a, miR-24, miR-25, miR-210, and miR-26a) [15–19] and beta-cell regulatory networks (miR-24, miR-148a, miR-200a, and miR-29a) [20]. Even more importantly we found quite a few miRNAs with yet unidentified function related to T1D as miR-152 and miR-30a-5p.

miRNAs are known to play a central role in posttranscriptional gene regulation and are involved in many cellular processes, such as differentiation, proliferation, and apoptosis [20]. miRNAs are detectable in cell-free circulation, that is, plasma and serum, and thus have been investigated as noninvasive biomarkers in several diseases and pathologic processes. In type 2 diabetes (T2D), which is characterized by chronic elevations of blood glucose levels and insulin resistance, one study identified a unique plasma miRNA signature for T2D, which included reduced levels of miR-15a, miR-20b, miR-21, miR-24, miR-126, miR-191, miR-197, miR-223, miR-320, miR-486 and elevated levels of miR-28-3p. Intriguingly, a reduction in the level of some of these miRNAs (miR-126, miR-15a, and miR-223) was already detectable years before the manifestation of diabetes [21]. Of these, only miR-24 overlaps with our observation. miR-24 is a regulator of apoptosis [16] and TGF*β* signaling [22] suggesting a role in inflammation which is important in both T1D and T2D development [23]. A study by Kong et al. [24] analyzed seven diabetes-related miRNA candidates in serum from newlydiagnosed T2D patients, prediabetic and healthy subjects and found miR-34a to be the strongest predictor of T2D. No overlap with the markers identified by Zampetaki et al. [21] was observed.

Current circulating biomarkers for T1D and residual beta-cell function are based on specific immunoglobulin (autoantibodies) and C-peptide measurements which are expensive and time consuming in a routine clinical setting. The development of new protein-based biomarkers is often rather cumbersome because of the complexity of protein composition in blood, the diversity of posttranslational modifications, the low abundance of many proteins, and challenges in developing high-sensitivity assays. 

The precise cellular release mechanisms of miRNA are largely unknown, and, therefore, the role of circulating miRNAs is not fully understood. However, miRNAs offer many features as attractive biomarkers: stability and evolutionary conservation, and because they can be detected by real-time PCR, assays can be highly sensitive and specific. 

Interestingly we found an association between miR-25 and better glycaemic control and residual beta-cell function. There have been several studies linking the miR-25 to different pathological conditions especially in cancer. Several reports associate high serum miR-25 with breast cancer, non-small-cell lung carcinoma and hepatocellular carcinoma [8, 25–27]. Furthermore, miR-25 has been found to be expressed in several malignant cell lines (ovarian cancer cell line and cholangiocarcinoma), where it is involved in regulation of apoptosis and cell proliferation by targeting proapoptotic proteins as Bim and Trail (TNF-related apoptosis inducing ligand) [17, 28]. A previous study in an experimental diabetic nephropathy model investigated the role of miRNAs in the regulation of NADPH expression during hyperglycaemia and found miR-25 was significantly reduced in kidney from these animals [18]. Our present findings of improved residual beta-cell function in patients with high level of miR-25 are in accordance with these results, suggesting a role of miR-25 on cell proliferation of the endocrine cells of the pancreas. This is also supported by the lack of association between miR-25 and the degree of autoimmunity (as assessed by presence of autoantibodies) in our study. The concordance between improved stimulated C-peptide and better HbA1c levels supports the potential role of this miRNA during disease progression in these children, and that is despite the limited number of individuals included in this study. 

This study provides suggestive evidence for the role of miRNAs as clinical applicable biomarkers in T1D. The association of miR-25 with improved glycaemic control and better residual beta-cell function may indicate that this miRNA could be an important biomarker that could be used during early and intensive management of newly diagnosed diabetes to improve blood glucose control and reduce microvascular complications. These findings should be confirmed in an independent study population; however, we are currently investigating the miR-25 levels in all patients from our study cohorts at different time points during disease progression. This study can serve as model for conceptualising the use of miRNAs as clinical relevant biomarkers in which they potentially will be used as beneficial predictors to evaluate clinically meaningful changes in intervention therapies designed to preserve/regenerate beta-cell function in new onset T1D. 

## 5. Conclusions

This study shows that 12 miRNAs have increased expression levels in children with new onset T1D compared to age-matched healthy controls. Furthermore, the residual beta-cell function and glycaemic control after 3 months of clinical disease associate with miR-25 expression level present soon after diagnosis. These findings indicate a potential role for miRNAs in the understanding of disease mechanisms at an early time point; this knowledge may in the future be translated into optimized and individualized diabetes management (bench-to-bedside) to the benefit of the patients.

## Figures and Tables

**Table 1:** List of miRNAs differentially expressed in sera from children and adolescents with newly diagnosed T1D compared to sera from age-matched controls.

Systematic miRNA name	ΔCt change	*P*_corrected_ value	Regulation T1D/Controls	Difference in following cohorts
hsa-miR-152	2.09	<0.0001	Up	Hvidoere/controls
hsa-miR-30a-5p	1.52	<0.0001	Up	Hvidoere/controls Danish/controls
hsa-miR-181a	2.30	<0.0001	Up	Hvidoere/controls Danish/controls
hsa-miR-24	2.25	<0.0001	Up	Hvidoere/controls
hsa-miR-148a	2.25	0.00015	Up	Hvidoere/controls
hsa-miR-210	1.65	0.00078	Up	Hvidoere/controls
hsa-miR-27a	2.79	0.00139	Up	Hvidoere/controls
hsa-miR-29a	2.39	0.00636	Up	Hvidoere/controls
hsa-miR-27b	1.13	0.00953	Up	Hvidoere/controls
hsa-miR-26a	1.31	0.01554	Up	Hvidoere/controls Danish/controls
hsa-miR-25	1.53	0.02013	Up	Hvidoere/controls Danish/controls
hsa-miR-200a	1.23	0.02957	Up	Hvidoere/controls

## Abbreviations

T1D: Type 1 diabetes
miRNA: microRNA
HDL: High-density lipoprotein
qRT-PCR: Quantitative real time polymerase chain reaction.
