# Erratum to “Circulating Levels of MicroRNA from Children with Newly Diagnosed Type 1 Diabetes and Healthy Controls: Evidence That miR-25 Associates to Residual Beta-Cell Function and Glycaemic Control during Disease Progression”

**DOI:** 10.1155/2012/672865

**Published:** 2012-12-02

**Authors:** Lotte B. Nielsen, Cheng Wang, Kaspar Sørensen, Claus H. Bang-Berthelsen, Lars Hansen, Marie-Louise M. Andersen, Philip Hougaard, Anders Juul, Chen-Yu Zhang, Flemming Pociot, Henrik B. Mortensen

**Affiliations:** ^1^Department of Pediatrics, Herlev Hospital, 2730 Herlev, Denmark; ^2^Faculty of Health Sciences, University of Copenhagen, 2200 Copenhagen, Denmark; ^3^Jiangsu Engineering Research Center for microRNA Biology and Biotechnology, State Key Laboratory of Pharmaceutical Biotechnology, School of Life Sciences, Nanjing University, Nanjing 210093, China; ^4^Department of Growth and Reproduction, Rigshospitalet and Faculty of Health Sciences, University of Copenhagen, 2100 Copenhagen, Denmark; ^5^Glostrup Research Institute, Glostrup Hospital and Center for Non-Coding RNA in Technology and Health, University of Copenhagen, 2600 Glostrup, Denmark; ^6^Department of Statistics, University of Southern Denmark, 5000 Odense, Denmark

The following sentence: Furthermore, miR-25 one month after diagnosis was positively associated with residual beta-cell function (*P* = 0.0037), and negatively associated with glycaemic control (HbA1c) (*P* = 0.0035) 3 months after disease onset, should replace this sentence in the abstract of the existing paper: Furthermore, we identified miR-25 as negatively associated with residual beta-cell function (est.: −0.12, *P* = 0.0037), and positively associated with glycaemic control (HbA1c) (est.: 0.11, *P* = 0.0035) 3 months after onset.

